# Supplementary material for: Enzymatic Electroanalytical Biosensor Based on Maramiellus colocasiae Fungus for Detection of Phytomarkers in Infusions and Green Tea Kombucha
Source: Biosensors (Basel). 2021 Mar 22;11(3):91. doi: 10.3390/bios11030091 (PMC8004623; doi:10.3390/bios11030091)
Supplement: Supplementary file 1 [file biosensors-11-00091-s001.pdf]

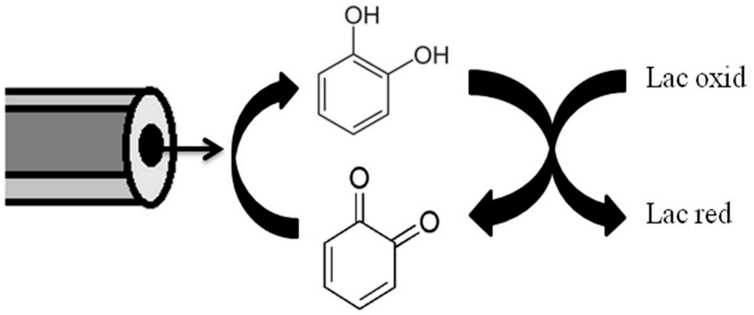

Figure S1. Redox enzymatic mechanism of the laccase with the catechol phenolic marker.

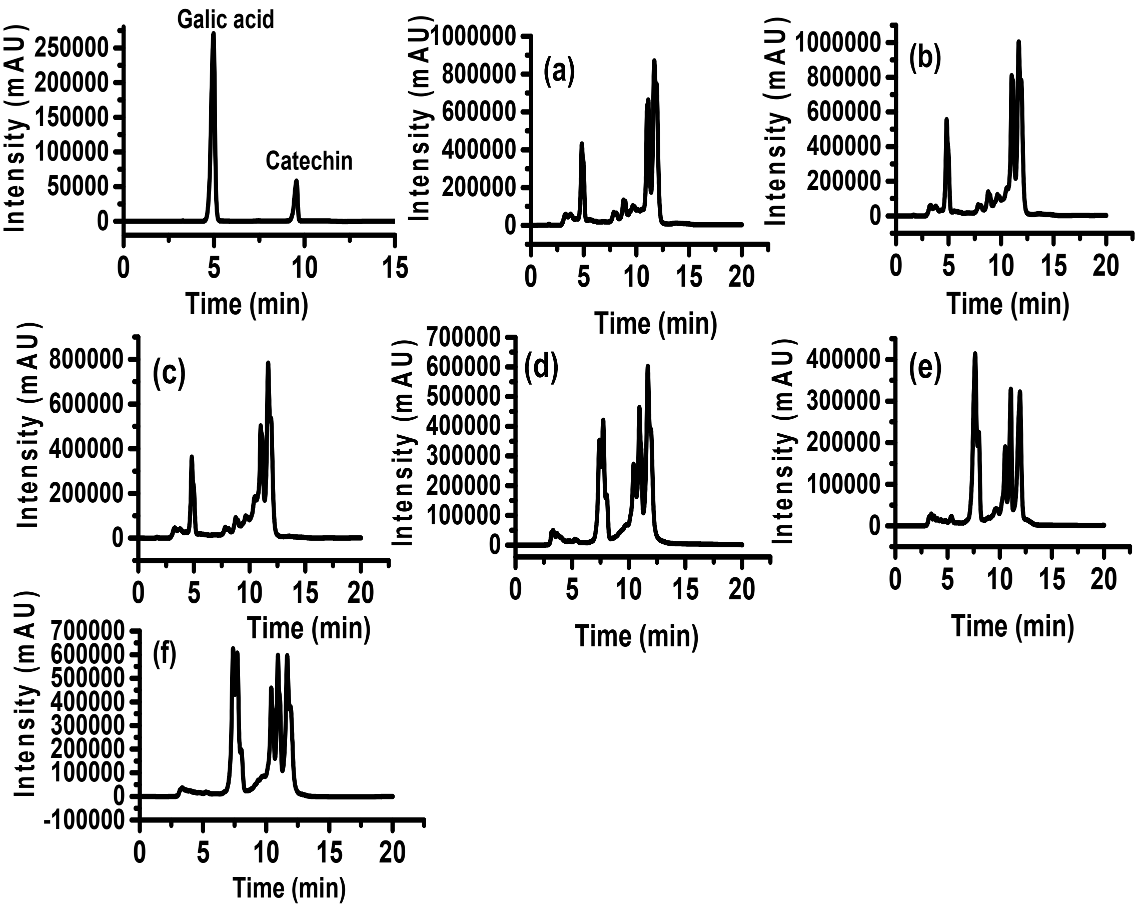

Figure S2. Evaluation of commercial green tea of different samples and brands of (**a**–**f**) by HPLC, infused for 2 min. Mobile phase 0.1% phosphoric acid: Methanol. Gradient elution mode, detection performed at 280 nm wavelength.
